# Treatment of Henoch Schonlein nephritis; new trends

**DOI:** 10.15171/jnp.2016.21

**Published:** 2016-07-28

**Authors:** Azar Nickavar

**Affiliations:** Department of Pediatric Nephrology, Iran University of Medical Sciences, Tehran, Iran

**Keywords:** Henoch-Schönlein nephritis, Chronic kidney disease, Endocapillary proliferation

**Implication for health policy/practice/research/medical education:**

Henoch-Schönlein purpura (HSP) is the most common small vessel vasculitis in children and adolescents with high incidence of renal involvement. Delayed and inappropriate management of severe Henoch-Schönlein nephritis is a major cause of chronic kidney disease. The most recent treatments of Henoch-Schönlein nephritis have been discussed in this editorial article.

Henoch-Schönlein purpura (HSP) was first described more than 200 years ago by Heberdon. It is the most common systemic vasculitis of childhood. Short and long term outcomes of HSP is generally favorable, with complete and spontaneous resolution of symptoms (1-3).

Renal involvement occurs in 40%-60% of pediatric patients within 4 to 6 weeks of the initial presentation (1). It is the major cause of mortality and morbidity in children with HSP (2) and prognosis is mainly dependent upon the severity of renal involvement (4).

Majority of patients with Henoch-Schönlein purpura nephritis (HSN) have a mild disease, presenting with hematuria and/or low-grade proteinuria, with a high recovery rate. A small percentage of patients present with nephrotic syndrome or renal function impairment (1,2).

A direct correlation has been suggested between the intensity of clinical manifestations, histopathologic grading, and renal outcome in HSN. There is a positive relationship between the severity of proteinuria, nephrotic syndrome and acute nephritis with both pathologic grading and scoring, particularly crescent formation, endocapillary proliferation, and tubular atrophy (2).

It has been suggested that older age at presentation, hypertension, increased serum creatinine, early onset nephritic, mixed nephritic–nephrotic syndrome at the onset of disease, extensive crescents, severity of glomerular necrosis and sclerosis, with tubulointerstitial damage are the major risk factors of renal impairment in these patients (2,5).

Chronic kidney disease occurs in almost 20% of children admitted to the tertiary care centers (4). About 1%-7% of unselected patients with HSN may progress to end-stage renal disease by 20 years after diagnosis (1,6). However, early diagnosis, along with appropriate treatment and timely management improve eventual renal outcome in these patients (3).

There is no consensus for the treatment of HSN (1) and the most effective treatment remains controversial (7). It has been suggested that early corticosteroids treatment may not prevent the development of HSN and should not be routinely recommended (8,9).

Renal biopsy or immunosuppressive treatments is not recommended in patients with mild renal symptoms such as microhematuria, mild proteinuria and normal renal function which needs to regular follow up for the early detection of renal deterioration (1,3).

Data on the treatment of severe HSN are controversial and scarce (10). KDIGO guidelines recommended early treatment with angiotensin converting enzyme inhibitors (ACE-I) or angiotensin receptor blockers (ARBs) in patients with persistent proteinuria (1), to improve long term renal outcome independent of histologic lesions (11). A 6-month course of corticosteroid therapy is recommended in those with persistent proteinuria and glomerular filtration rate >50 mL/min per 1.73 m^2^.

Early immunosuppressive therapy with high dose corticosteroids, cyclophosphamide, azathioprine (1) calcineurin inhibitors (12), mycophenolate mofetil (MMF) (13), rituximab (3), mizoribine or methotrexate (1) have been suggested in patients with significant kidney involvement (proteinuria in nephrotic range and/or progressive kidney impairment) (3). The main objectives of immunosuppressive treatments are prevention of irreversible glomerular fibrosis with increasing proteinuria and improvement long term renal outcome (5,14).

In addition, combined immunosuppressive treatments with warfarin, dipyridamole, acetylsalicylic acid, tonsillectomy for eradication of chronic bacterial infections, hemoperfusion for elimination of immune mediators (3), intravenous immunoglobulin (IVIG) (5), plasma exchange for removal of inflammatory and procoagulant circulating complexes, antiplatelet drugs and vitamins have been suggested for treatment of HSN. However, there is not enough evidence about increased efficacy of immune suppression or multiple-drug treatment in children with severe HSN (1).

## Conflicts of interest

The author declared no competing interests.

## Author’s contribution

AN was the single author of the manuscript.

## Funding/Support

None
